# Supplementary material for: Extended stop codon context predicts nonsense codon readthrough efficiency in human cells
Source: Nat Commun. 2024 Mar 20;15:2486. doi: 10.1038/s41467-024-46703-z (PMC10954755; doi:10.1038/s41467-024-46703-z)
Supplement: Supplementary file 1 — Supplementary Information [file 41467_2024_46703_MOESM1_ESM.pdf]

Extended stop codon context predicts nonsense codon readthrough efficiency in human cells

Supplementary Information

Page #

Supplementary Fig. 1. Random forest model performance metrics..2

Supplementary Fig. 2. SCR mRNAs..3

Supplementary Fig. 3. Frequency of stop codon and nt +4 in each sample...............................5

Supplementary Fig. 4. Readthrough efficiency increases with 3'-UTR length............................7

Supplementary Fig. 5. The effect of 3'-UTR length on readthrough efficiency in context with other mRNA features..8

Supplementary Fig. 6. Measurement of PTC readthrough by Dual-Luciferase assay................9

Supplementary Fig. 7. Readthrough efficiency prediction using limited mRNA features...........12

Supplementary Fig. 8. Stop codon context of *CFTR* PTC alleles..13

Supplementary References 15

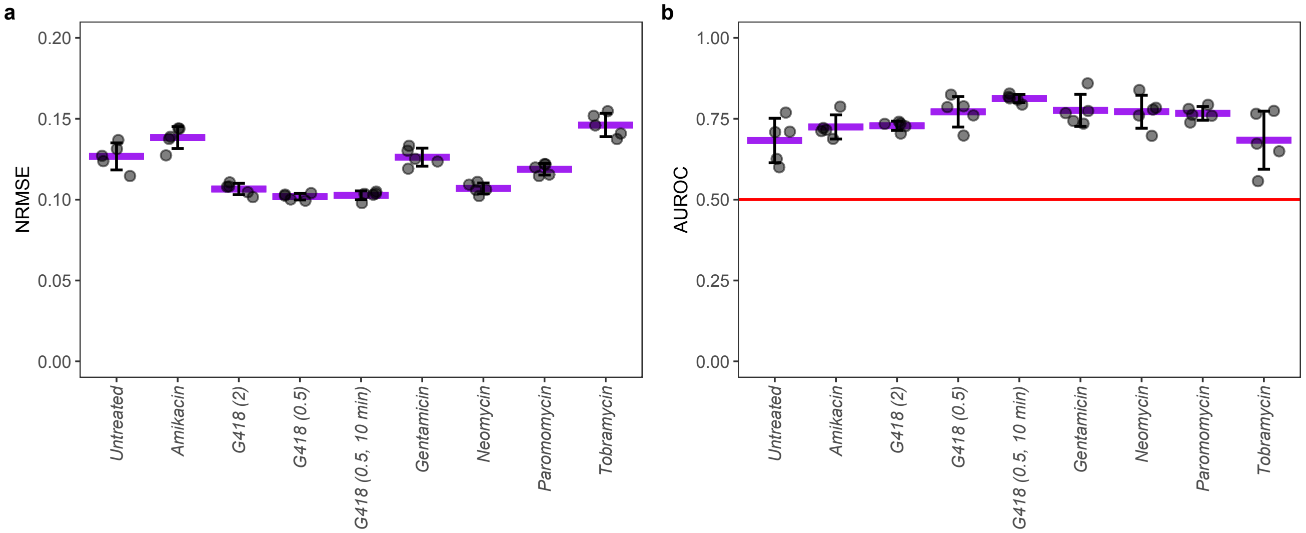

Supplementary Fig. 1. Random forest model performance metrics.
a Normalized Root Mean Square Error (NRMSE) shows prediction errors of random forest regression models. **b** The area under the receiver operating characteristic (AUROC) reports the capability of the model to distinguish between "high" and "low" readthrough mRNAs, which is higher than random chance (0.5, red horizontal line) in all samples. For both metrics, the values were average (purple line) ± standard deviation (error bar) of 5-fold cross-validation (n = 5). Source data are provided as a Source Data file.

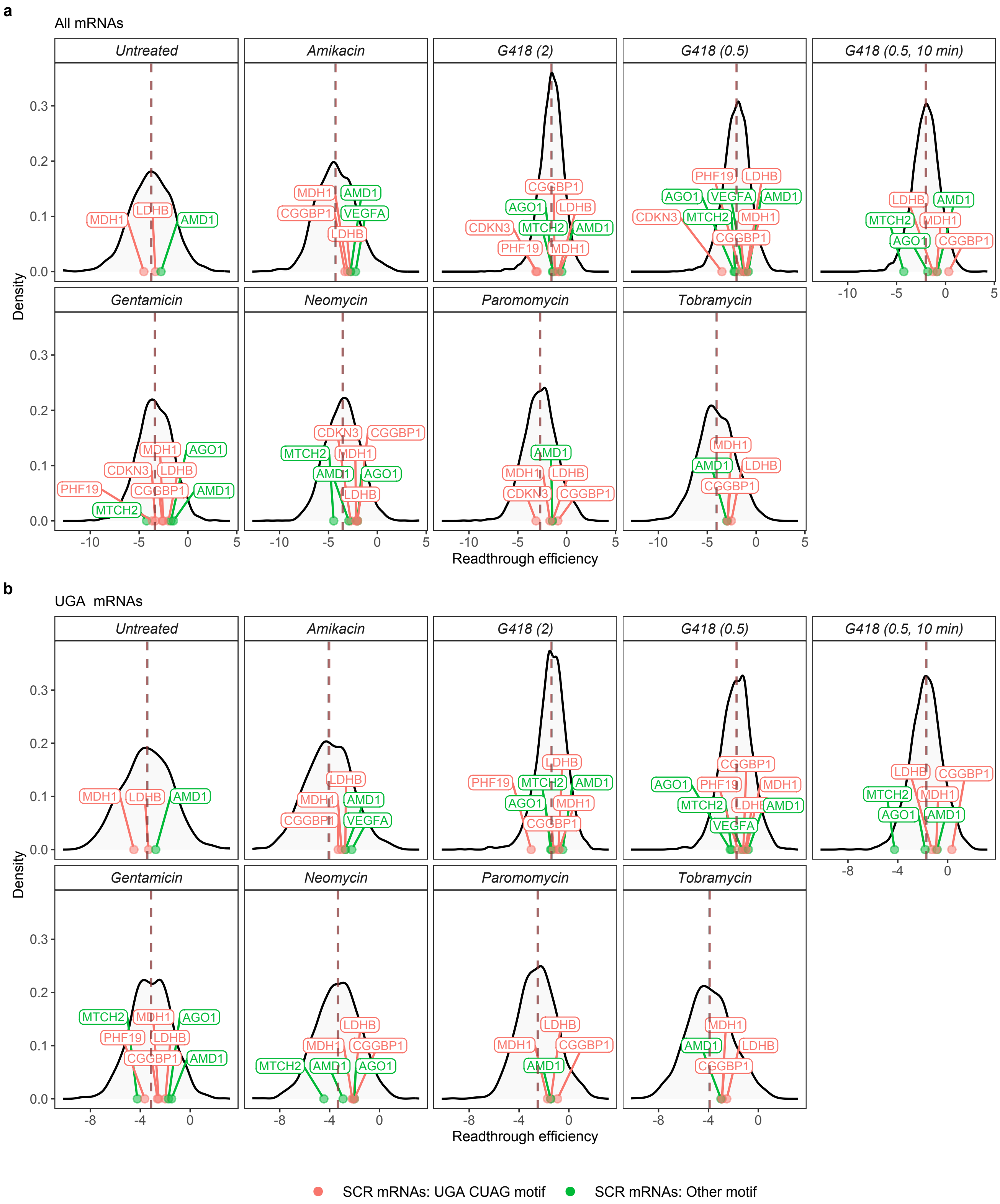
a
All mRNAs
Untreated
Amikacin
G418 (2)
G418 (0.5)
G418 (0.5, 10 min)
Gentamicin
Neomycin
Paromomycin
Tobramycin
Density
Readthrough efficiency
b
UGA mRNAs
Untreated
Amikacin
G418 (2)
G418 (0.5)
G418 (0.5, 10 min)
Gentamicin
Neomycin
Paromomycin
Tobramycin
Density
Readthrough efficiency
SCR mRNAs: UGA CUAG motif
SCR mRNAs: Other motif

Supplementary Fig. 2. SCR mRNAs.

Readthrough efficiency of mRNAs known to undergo programmed stop codon readthrough[1,2] (SCR mRNAs, red and green) relative to the distribution of readthrough efficiency of all mRNAs in the sample (**a**) or only UGA-containing mRNAs (**b**). The mRNA names are labeled. Mean and median readthrough efficiency are indicated by brown and grey dashed lines, respectively (In most cases, they overlap visually). Source data are provided as a Source Data file.

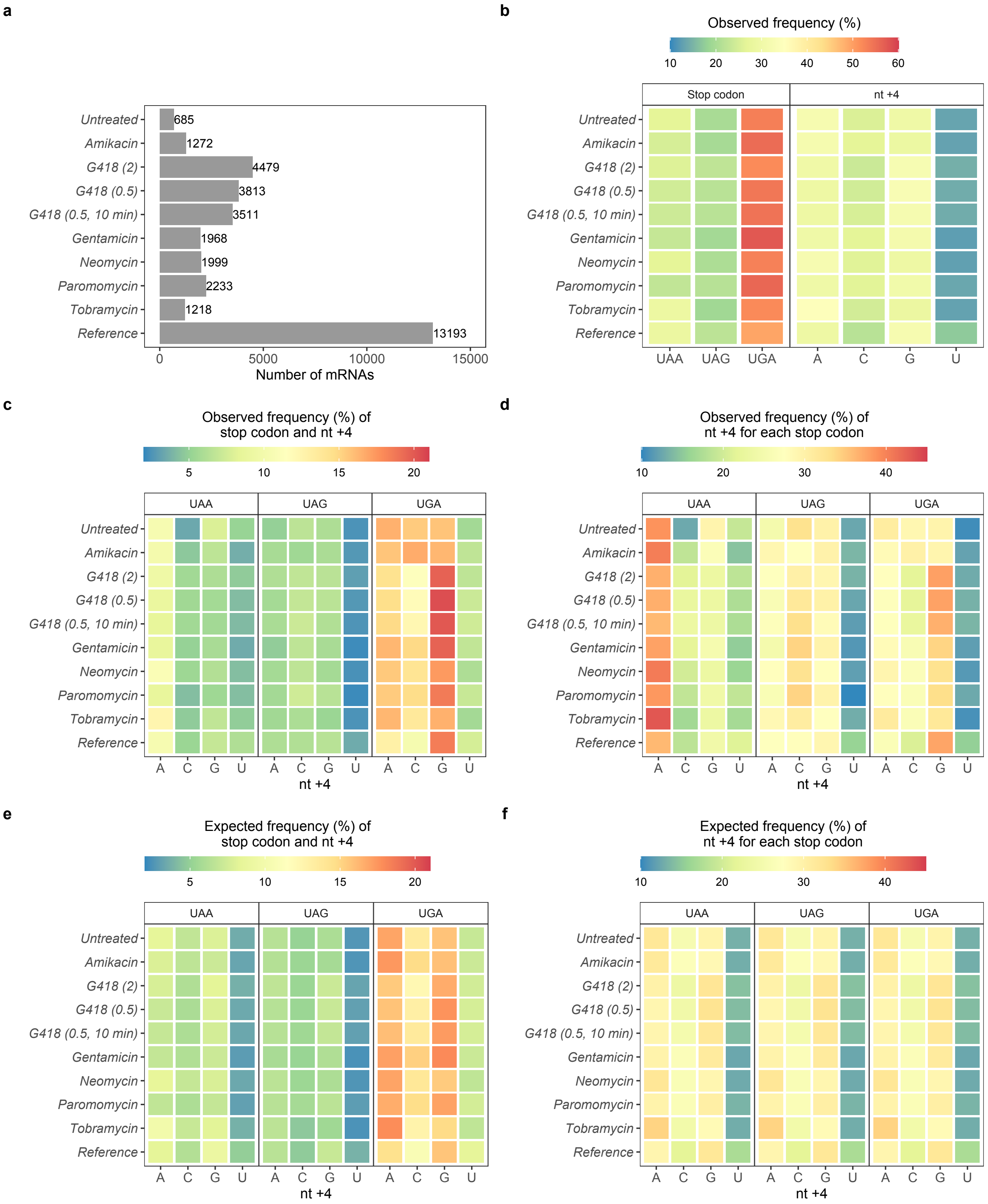
a
Untreated
Amikacin
G418 (2)
G418 (0.5)
G418 (0.5, 10 min)
Gentamicin
Neomycin
Paromomycin
Tobramycin
Reference
685
1272
4479
3813
3511
1968
1999
2233
1218
13193
0
5000
10000
15000
Number of mRNAs
b
Observed frequency (%)
10 20 30 40 50 60
Stop codon
nt +4
UAA UAG UGA
A C G U
c
Observed frequency (%) of stop codon and nt +4
5 10 15 20
UAA UAG UGA
nt +4
d
Observed frequency (%) of nt +4 for each stop codon
10 20 30 40
e
Expected frequency (%) of stop codon and nt +4
5 10 15 20
f
Expected frequency (%) of nt +4 for each stop codon
10 20 30 40

Supplementary Fig. 3. Frequency of stop codon and nt +4 in each sample.

a Number of mRNAs with detectable readthrough in ribosome profiling data of HEK293T cells treated with different aminoglycosides. Reference sample consists of all mRNAs detected by ribosome profiling in all samples combined regardless of whether readthrough was detectable or not. **b** Percentage of stop codon (left) and nt +4 (right) observed in each sample. **c** Percentage of stop codon and nt +4 as quadruplets observed in each sample. **d** Percentage of nt +4 for each stop codon observed in each sample. **e** Expected percentage of stop codon and nt +4 as quadruplets in each sample if nts +4 were distributed evenly among the stop codons. **f** Expected percentage of nt +4 for each stop codon in each sample if nts +4 were distributed evenly among the stop codons. Source data are provided as a Source Data file.

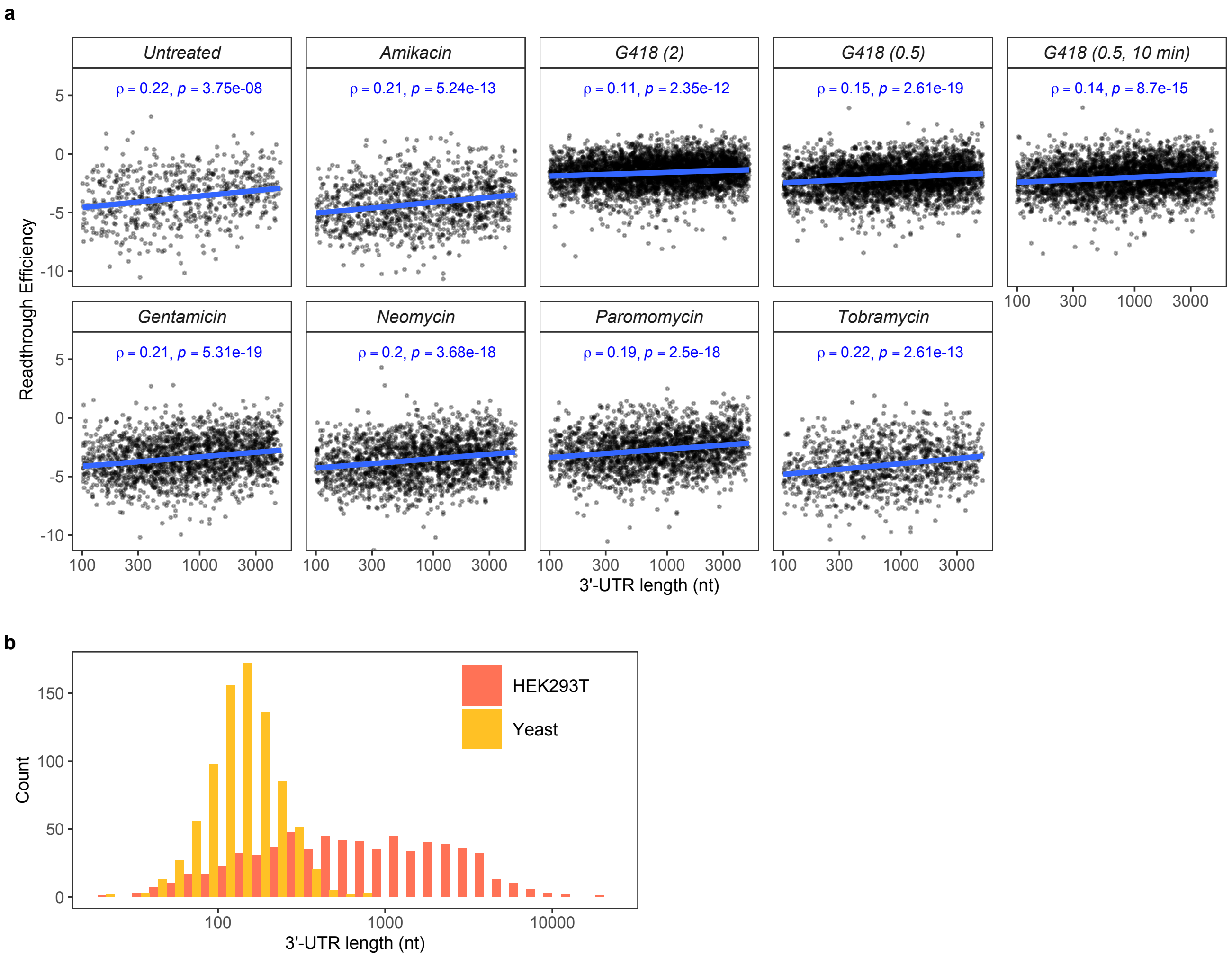

Supplementary Fig. 4. Readthrough efficiency increases with 3'-UTR length.
a Readthrough efficiency vs. 3'-UTR length for mRNAs with 3'-UTR lengths longer than 100 nt but shorter than 5,000 nt. Two-tailed Spearman's correlation coefficient (ρ) and the associated p-value are reported for each sample. **b** Distribution of 3'-UTR lengths for mRNAs that have UTR annotations in untreated HEK293T data set (pink)[3] and WT yeast data set (yellow) (*SUP45*, 25 °C from Mangkalaphiban et al. 2021)[4]. Source data are provided as a Source Data file.

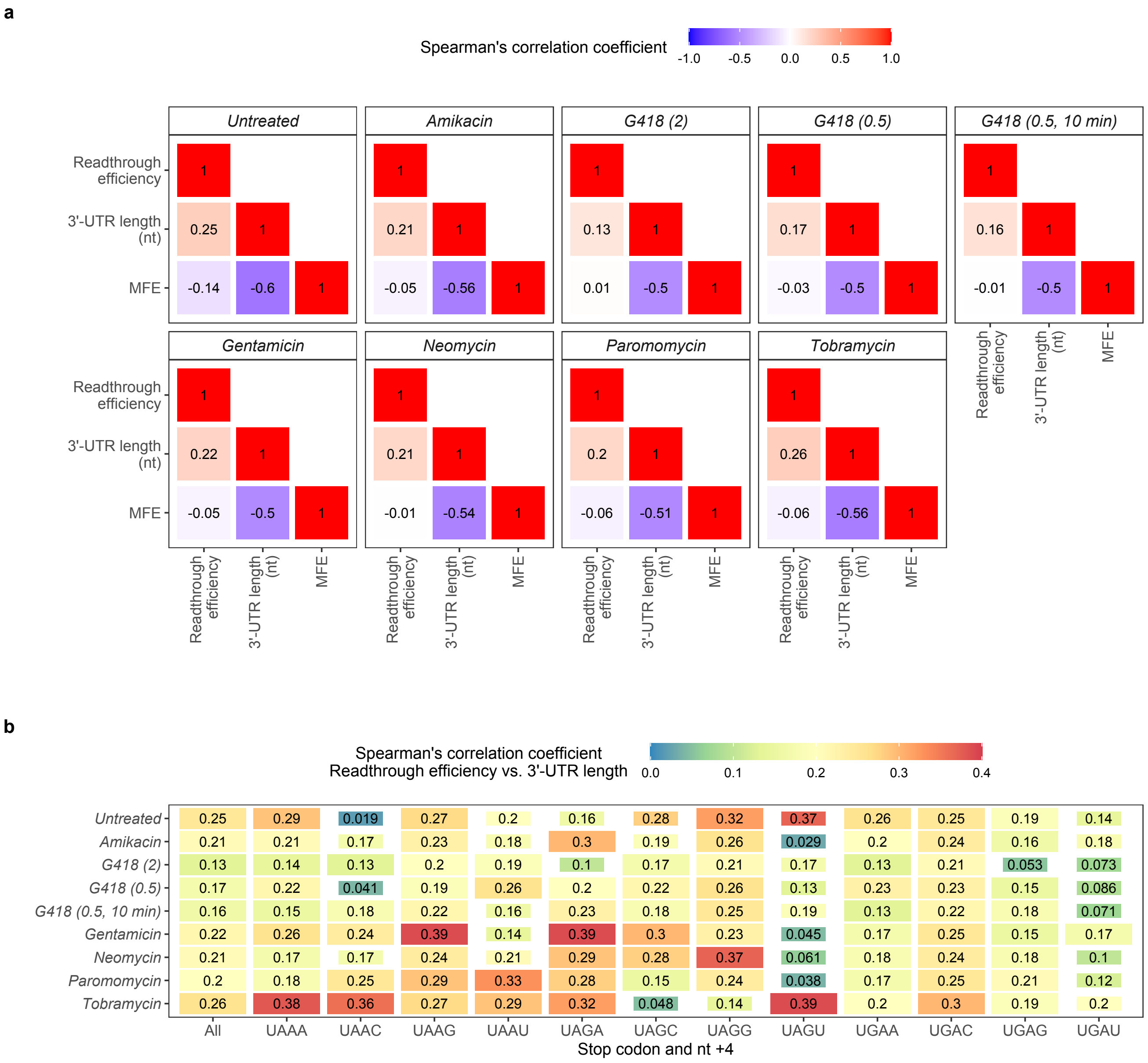

Supplementary Fig. 5. The effect of 3'-UTR length on readthrough efficiency in context with other mRNA features.

a Correlation matrix showing pairwise Two-tailed Spearman's. correlation efficient (ρ) for readthrough efficiency vs. 3'-UTR length (same data as Fig. 3), readthrough efficiency vs. minimum free energy (MFE) in the 3'-UTR, and 3'-UTR length vs. MFE for each sample. **b** Readthrough efficiency vs. 3'-UTR length for all mRNAs ("All", same as Fig. 3) or mRNAs having certain combinations of stop codon and nt +4 in the sample. Two-tailed Spearman's correlation coefficient (ρ) is represented by the color spectrum as well as labeled. Larger tile size indicates significant correlation ($p < 0.05$) and smaller tile size indicates insignificance ($p \geq 0.05$). Source data, the exact p-values, and number of data points (n) in each group are provided as a Source Data file.

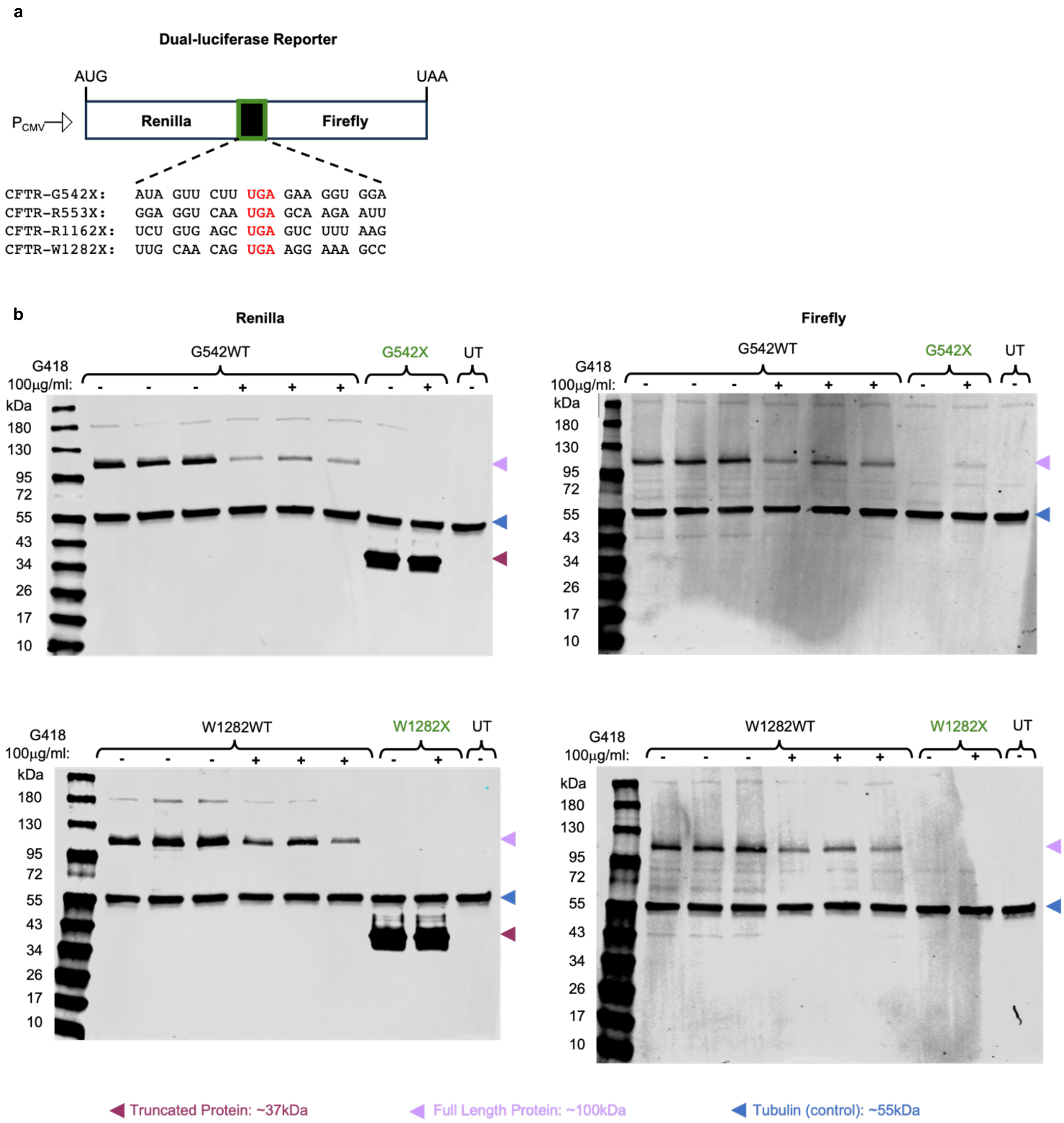
a
Dual-luciferase Reporter
AUG
UAA
PCMV
Renilla
Firefly
CFTR-G542X: AUA GUU CUU UGA GAA GGU GGA
CFTR-R553X: GGA GGU CAA UGA GCA AGA AUU
CFTR-R1162X: UCU GUG AGC UGA GUC UUU AAG
CFTR-W1282X: UUG CAA CAG UGA AGG AAA GCC
b
Renilla
Firefly
G418 100µg/ml:
G542WT
G542X
UT
W1282WT
W1282X
kDa
180
130
95
72
55
43
34
26
17
10
Truncated Protein: ~37kDa
Full Length Protein: ~100kDa
Tubulin (control): ~55kDa

c

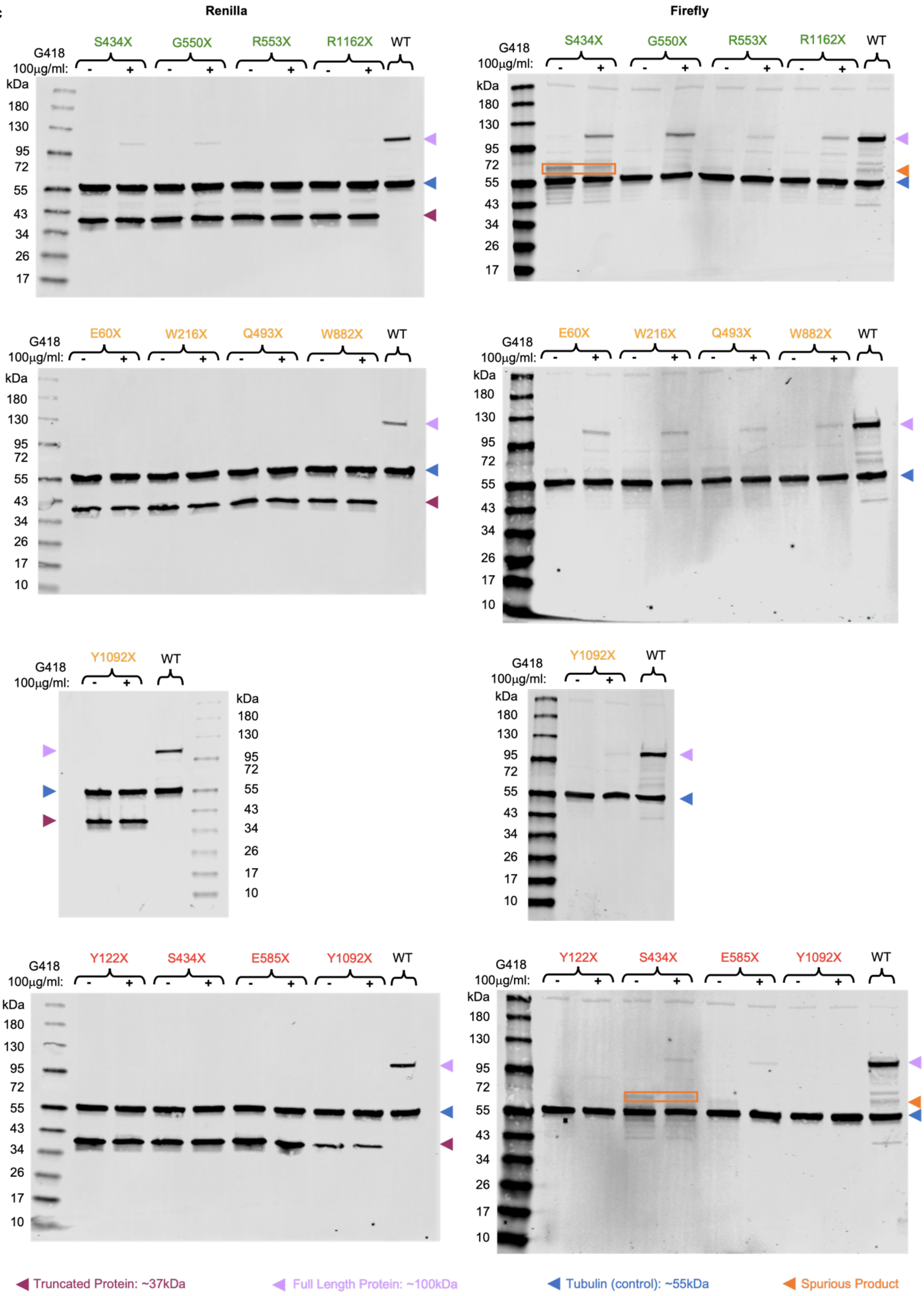
Renilla
Firefly
G418 100µg/ml:
S434X
G550X
R553X
R1162X
WT
E60X
W216X
Q493X
W882X
Y1092X
Y122X
E585X
kDa
180
130
95
72
55
43
34
26
17
10
Truncated Protein: ~37kDa
Full Length Protein: ~100kDa
Tubulin (control): ~55kDa
Spurious Product

Supplementary Fig. 6. Measurement of PTC readthrough by Dual-Luciferase assay.
a Schematic of dual-luciferase (Dual-Luc) reporter used to measure readthrough of *CFTR* PTC alleles. PTC context (stop codon and flanking 3 codons) was inserted between *Renilla* and firefly luciferase genes. Readthrough efficiency is firefly signal normalized to *Renilla* signal in percentage. **b and c.** Western blotting validation of truncated (~37kDa) and full-length readthrough (~100kDa) products of Dual-Luc constructs from cell lysates using anti-*Renilla* antibody (left) or anti-firefly antibody (right). UT = un-transfected control, WT = G542 WT. Spurious products that may result in luciferase activity are boxed in orange.

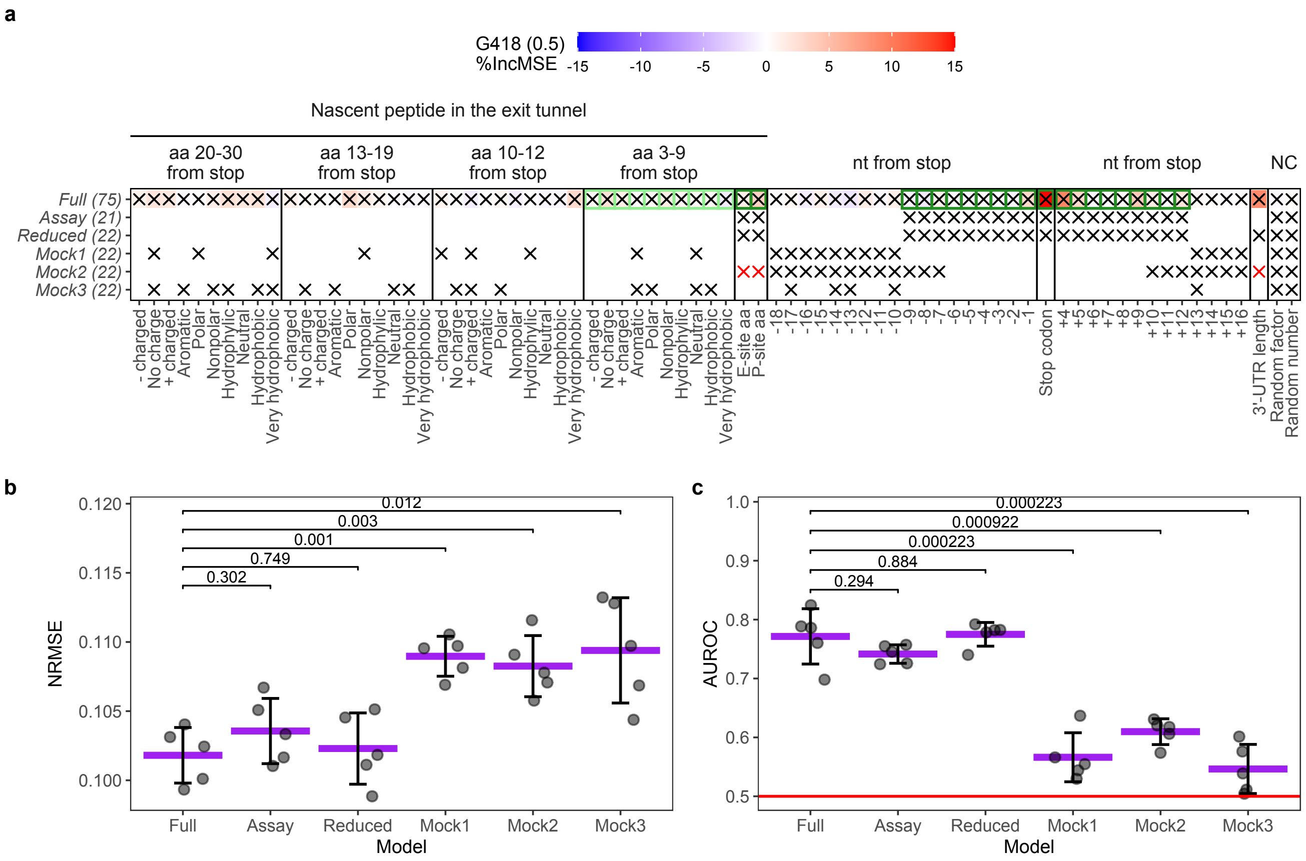

Supplementary Fig. 7. Readthrough efficiency prediction using limited mRNA features. **a** Comparison of mRNA features important in readthrough efficiency prediction and those captured in PTC context in Dual-Luc assay. Feature importance scores are from random forest regression model (%IncMSE) trained on G418 (0.5) data ("Full"), as in Fig. 1a. mRNA features varied between different PTC alleles in Dual-Luc assay are in dark green boxes, while those that were combinations of PTC context and reporter sequence are in light green boxes. mRNA features used in different random forest models (Y-axis) are indicated by black "X". Number of mRNA features used in each model excluding the two negative controls, NC) is indicated in parentheses. Compared to the "Full" model, the "Reduced" model omits features unimportant in readthrough efficiency prediction but also maintains minimal features used in Dual-Luc assay. The three "Mock" models serve as controls for the "Reduced" model, having the same number of features as the "Reduced" model. Red "X" for "Mock2" model are features not in the original "Full" model at all but have data type and data range similar to the indicated feature to control for model behavior: identities of P- and E-site amino acids were replaced by amino acids 5 and 10 codons upstream of stop codon; 3'-UTR length was replaced by 5'-UTR length. The "Assay" model represents minimal features captured in Dual-Luc assay. **b and c.** Performance metrics for each random forest model from the regression approach, NRMSE (b), and classification approach, AUROC (c). For both metrics, the values were average (purple line) ± standard deviation (error bar) of 5-fold cross-validation (n = 5). Two-tailed Student's t-test was used to compare each model to the "Full" model, with Benjamini-Hochberg method for multiple-testing correction. Additional abbreviations: aa = amino acid; nt = nucleotide. Source data are provided as a Source Data file.

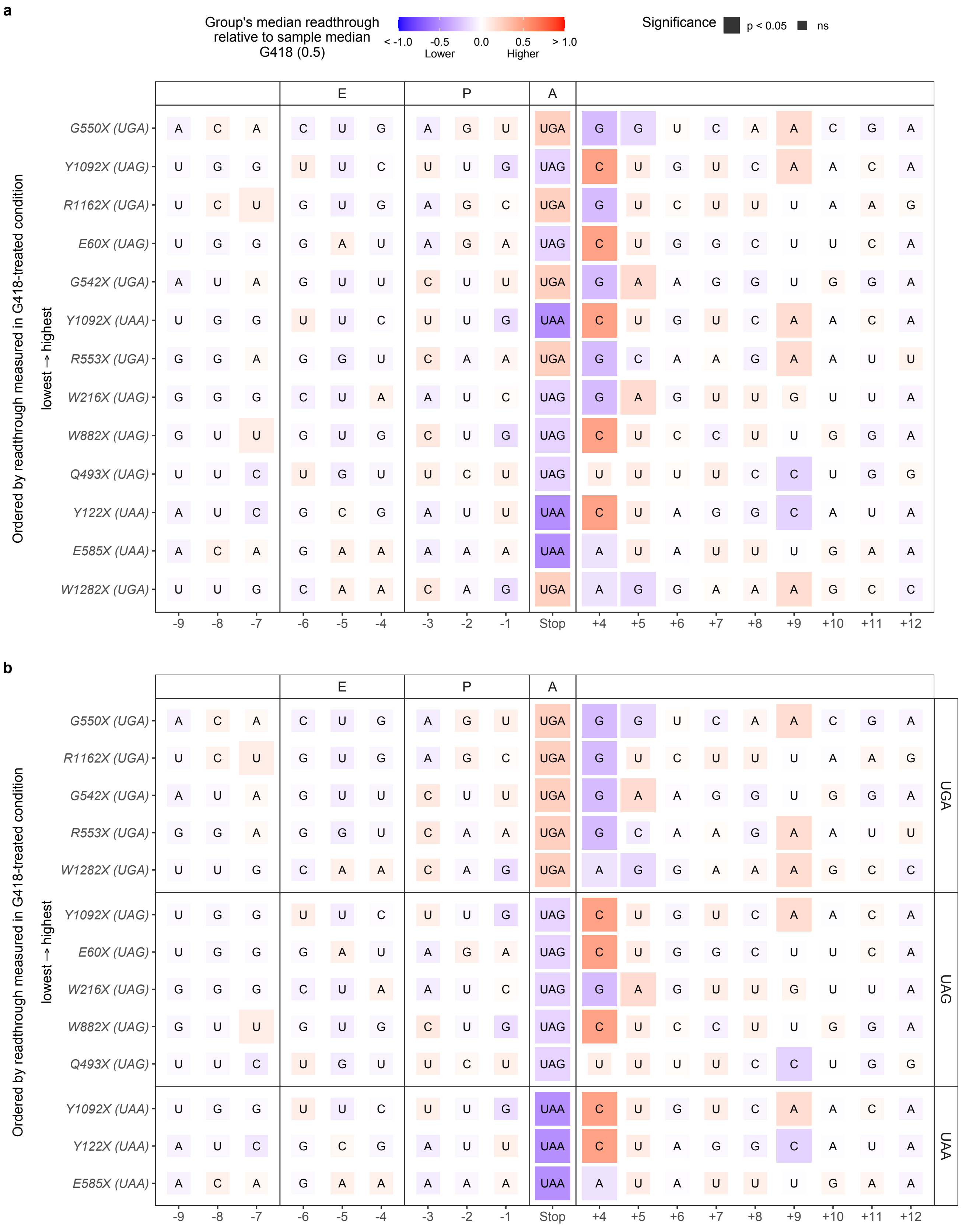
a
Group's median readthrough relative to sample median G418 (0.5)
< -1.0 -0.5 0.0 0.5 > 1.0
Lower Higher
Significance p < 0.05 ns
E P A
Ordered by readthrough measured in G418-treated condition
lowest → highest
G550X (UGA) A C A C U G A G U UGA G G U C A A C G A
Y1092X (UAG) U G G U U C U U G UAG C U G U C A A C A
R1162X (UGA) U C U G U G A G C UGA G U C U U U A A G
E60X (UAG) U G G G A U A G A UAG C U G G C U U C A
G542X (UGA) A U A G U U C U U UGA G A A G G U G G A
Y1092X (UAA) U G G U U C U U G UAA C U G U C A A C A
R553X (UGA) G G A G G U C A A UGA G C A A G A A U U
W216X (UAG) G G G C U A A U C UAG G A G U U G U U A
W882X (UAG) G U U G U G C U G UAG C U C C U U G G A
Q493X (UAG) U U C U G U U C U UAG U U U U C C U G G
Y122X (UAA) A U C G C G A U U UAA C U A G G C A U A
E585X (UAA) A C A G A A A A A UAA A U A U U U G A A
W1282X (UGA) U U G C A A C A G UGA A G G A A A G C C
-9 -8 -7 -6 -5 -4 -3 -2 -1 Stop +4 +5 +6 +7 +8 +9 +10 +11 +12
b
E P A
Ordered by readthrough measured in G418-treated condition
lowest → highest
G550X (UGA) A C A C U G A G U UGA G G U C A A C G A
R1162X (UGA) U C U G U G A G C UGA G U C U U U A A G
G542X (UGA) A U A G U U C U U UGA G A A G G U G G A
R553X (UGA) G G A G G U C A A UGA G C A A G A A U U
W1282X (UGA) U U G C A A C A G UGA A G G A A A G C C
UGA
Y1092X (UAG) U G G U U C U U G UAG C U G U C A A C A
E60X (UAG) U G G G A U A G A UAG C U G G C U U C A
W216X (UAG) G G G C U A A U C UAG G A G U U G U U A
W882X (UAG) G U U G U G C U G UAG C U C C U U G G A
Q493X (UAG) U U C U G U U C U UAG U U U U C C U G G
UAG
Y1092X (UAA) U G G U U C U U G UAA C U G U C A A C A
Y122X (UAA) A U C G C G A U U UAA C U A G G C A U A
E585X (UAA) A C A G A A A A A UAA A U A U U U G A A
UAA
-9 -8 -7 -6 -5 -4 -3 -2 -1 Stop +4 +5 +6 +7 +8 +9 +10 +11 +12

Supplementary Fig. 8. Stop codon context of *CFTR* PTC alleles.
a Nucleotide sequences of PTC alleles colored at individual nucleotide position (X-axis) by the effects of nucleotide identity on readthrough efficiency in G418 (0.5) sample as in Fig. 2a. PTC alleles are ordered by readthrough efficiency measured by Dual-Luc assay in G418-treated condition (Y-axis). **b** As in a, but grouped by stop codon identity.

Supplementary References

1. Loughran, G. *et al.* Stop codon readthrough generates a C-terminally extended variant of the human vitamin D receptor with reduced calcitriol response. *Journal of Biological Chemistry* **293**, 4434–4444 (2018).

2. Manjunath, L. E., Singh, A., Som, S. & Eswarappa, S. M. Mammalian proteome expansion by stop codon readthrough. *WIREs RNA* e1739 (2022) doi:10.1002/wrna.1739.

3. Wangen, J. R. & Green, R. Stop codon context influences genome-wide stimulation of termination codon readthrough by aminoglycosides. *eLife* **9**, e52611 (2020).

4. Mangkalaphiban, K. *et al.* Transcriptome-wide investigation of stop codon readthrough in Saccharomyces cerevisiae. *PLoS Genet* **17**, e1009538 (2021).
