# Supplementary material for: Extended stop codon context predicts nonsense codon readthrough efficiency in human cells
Source: Nat Commun. 2024 Mar 20;15:2486. doi: 10.1038/s41467-024-46703-z (PMC10954755; doi:10.1038/s41467-024-46703-z)
Supplement: Supplementary file 3 — Description of Additional Supplementary Files [file 41467_2024_46703_MOESM3_ESM.pdf]

Description of Additional Supplementary Files

File Name: Supplementary Data 1
Description: This file contains raw firefly and *Renilla* luciferase signals as well as the firefly/*Renilla* ratios for 15 h*CFTR* PTC reporters.

File Name: Supplementary Data 2
Description: This file contains plasmids and oligonucleotide sequence information used to construct 15 dual-luciferase reporters containing different h*CFTR* PTC contexts.
