# Supplementary material for: Extended stop codon context predicts nonsense codon readthrough efficiency in human cells
Source: Nat Commun. 2024 Mar 20;15:2486. doi: 10.1038/s41467-024-46703-z (PMC10954755; doi:10.1038/s41467-024-46703-z)
Supplement: Supplementary file 6 — Reporting Summary [file 41467_2024_46703_MOESM6_ESM.pdf]

nature portfolio

Corresponding author(s): Allan Jacobson

Last updated by author(s): 2024/02/23

# Reporting Summary

Nature Portfolio wishes to improve the reproducibility of the work that we publish. This form provides structure for consistency and transparency in reporting. For further information on Nature Portfolio policies, see our Editorial Policies and the Editorial Policy Checklist.

## Statistics

For all statistical analyses, confirm that the following items are present in the figure legend, table legend, main text, or Methods section.

| n/a | Confirmed | |
|---|---|---|
| ☐ | ☒ | The exact sample size (*n*) for each experimental group/condition, given as a discrete number and unit of measurement |
| ☐ | ☒ | A statement on whether measurements were taken from distinct samples or whether the same sample was measured repeatedly |
| ☐ | ☒ | The statistical test(s) used AND whether they are one- or two-sided<br>*Only common tests should be described solely by name; describe more complex techniques in the Methods section.* |
| ☒ | ☐ | A description of all covariates tested |
| ☐ | ☒ | A description of any assumptions or corrections, such as tests of normality and adjustment for multiple comparisons |
| ☐ | ☒ | A full description of the statistical parameters including central tendency (e.g. means) or other basic estimates (e.g. regression coefficient) AND variation (e.g. standard deviation) or associated estimates of uncertainty (e.g. confidence intervals) |
| ☐ | ☒ | For null hypothesis testing, the test statistic (e.g. *F*, *t*, *r*) with confidence intervals, effect sizes, degrees of freedom and *P* value noted<br>*Give P values as exact values whenever suitable.* |
| ☒ | ☐ | For Bayesian analysis, information on the choice of priors and Markov chain Monte Carlo settings |
| ☒ | ☐ | For hierarchical and complex designs, identification of the appropriate level for tests and full reporting of outcomes |
| ☐ | ☒ | Estimates of effect sizes (e.g. Cohen's *d*, Pearson's *r*), indicating how they were calculated |

*Our web collection on statistics for biologists contains articles on many of the points above.*

## Software and code

Policy information about availability of computer code

| | |
|---|---|
| Data collection | The R package biomaRt (v 2.52.0) was used to download sequences of spliced mRNAs from Ensembl. |
| Data analysis | Analyses were performed in the R programming environment version 3.6 on the UMass high performance computing cluster (HPCC) and version 4.2 on macOS Monterey 12.6.3. The following R packages were used with R version 3.6: caret (v 6.0-86) and randomForest (v 4.6-14). The following R packages were used with R version 4.2: readxl (v 1.4.0), data.table (v 1.14.2), dplyr (v 1.0.8), reshape2 (v 1.4.4), biomaRt (v 2.52.0), randomForest (v 4.7-1), caret (v 6.0-92), Biostrings (v 2.64.0), seqinr (v 4.2-8), rstatix (v 0.7.0), ggplot2 (v 3.4.0), ggpubr (v 0.4.0), ggh4x (v 0.2.3), ggrepel (v 0.9.1), scales (v 1.2.1), patchwork (v 1.1.1), and Cairo (v 1.5-15). |

For manuscripts utilizing custom algorithms or software that are central to the research but not yet described in published literature, software must be made available to editors and reviewers. We strongly encourage code deposition in a community repository (e.g. GitHub). See the Nature Portfolio guidelines for submitting code & software for further information.

# Data

Policy information about availability of data

All manuscripts must include a data availability statement. This statement should provide the following information, where applicable:

- Accession codes, unique identifiers, or web links for publicly available datasets
- A description of any restrictions on data availability
- For clinical datasets or third party data, please ensure that the statement adheres to our policy

Readthrough efficiency data for HEK293T cells treated with different aminoglycosides were downloaded from Wangen and Green, eLife (2020), Fig. 2 – source data 1 (https://cdn.elifesciences.org/articles/52611/elife-52611-fig2-data1-v2.xlsx) and log2-transformed for all analyses. Sequences of spliced mRNAs were downloaded from Ensembl using R package biomaRt according to Ensembl Transcript ID provided in Wangen and Green, eLife (2020) Fig. 2 – source data 1.
Source data are provided with this paper. Raw luciferase signals and the firefly/Renilla ratios are provided in Supplementary Data 1. All processed data and each figure's source data are available in the Source Data File and without restriction at https://github.com/Jacobson-Lab/AG_readthrough (DOI: 10.5281/zenodo.10698037). Databases employed in this study include YeastMine (https://yeastmine.yeastgenome.org/yeastmine/begin.do) and Ensembl (https://useast.ensembl.org/index.html).

# Research involving human participants, their data, or biological material

Policy information about studies with human participants or human data. See also policy information about sex, gender (identity/presentation), and sexual orientation and race, ethnicity and racism.

| | |
|---|---|
| Reporting on sex and gender | NA |
| Reporting on race, ethnicity, or other socially relevant groupings | NA |
| Population characteristics | NA |
| Recruitment | NA |
| Ethics oversight | NA |

Note that full information on the approval of the study protocol must also be provided in the manuscript.

# Field-specific reporting

Please select the one below that is the best fit for your research. If you are not sure, read the appropriate sections before making your selection.

☒ Life sciences ☐ Behavioural & social sciences ☐ Ecological, evolutionary & environmental sciences

For a reference copy of the document with all sections, see nature.com/documents/nr-reporting-summary-flat.pdf

# Life sciences study design

All studies must disclose on these points even when the disclosure is negative.

**Sample size**

For readthrough efficiency data derived from ribosome profiling experiments, all mRNAs with reads mapped to them were initially considered for analyses. mRNAs with reads too sparse in the coding region, 3'-UTR region, or both (as described in the Methods section) were excluded because their readthrough efficiency values would be zero or unreliable, skewing downstream analyses. The number of mRNAs in each sample is provided in Supplementary Data Fig. 3a.

For CFTR PTC reporters, 8 alleles that are found most commonly in CF patients were chosen due to clinical relevance. An additional 7 alleles that are less prevalent in CF patients were added for diversity in the set of nonsense mutations analyzed, thereby maximizing the range of readthrough prediction. This set of 15 alleles, studied with and without G418 treatment, was at the limit of our technical abilities.

**Data exclusions**

For readthrough efficiency data derived from ribosome profiling experiments, all mRNAs with reads mapped to them were initially considered for analyses. mRNAs with reads too sparse in the coding region, 3'-UTR region, or both (as described in the Methods section) were excluded because their readthrough efficiency values would be zero or unreliable, skewing downstream analyses. The number of mRNAs in each sample is provided in Supplementary Data Fig. 3a.

Dual-luciferase assay measurements of 2 alleles (S434X UGA and UAA) were excluded from further analyses because their western blot validations showed spurious products that interfered with luciferase signal measurement. Outliers within each set of replicate wells were defined as replicates that made standard deviation/average (SD/AVE) % > 25%. They were excluded from AVE and SD calculations and indicated in red font in Supplementary Data 1.

**Replication**

For dual-lucfierase assay, 4-7 independent experiments were carried out for each CFTR PTC allele. Outliers within each set of replicate wells

| | |
|---|---|
| Replication | were defined as replicates that made standard deviation/average (SD/AVE) % > 25%. They were excluded from AVE and SD calculations and indicated in red font in Supplementary Data 1. |
| Randomization | Randomization was performed as part of the random forest algorithm. |
| Blinding | Individuals performing dual-luc readthrough assays were blinded from the machine learning predictions of readthrough efficiency. Vice versa, individuals performing machine learning predictions of readthrough were blinded from readthrough assay results. Only after the assays were complete that both parties were made aware of both results. |

# Reporting for specific materials, systems and methods

We require information from authors about some types of materials, experimental systems and methods used in many studies. Here, indicate whether each material, system or method listed is relevant to your study. If you are not sure if a list item applies to your research, read the appropriate section before selecting a response.

## Materials & experimental systems

| n/a | Involved in the study |
|---|---|
| ☐ | ☒ Antibodies |
| ☐ | ☒ Eukaryotic cell lines |
| ☒ | ☐ Palaeontology and archaeology |
| ☒ | ☐ Animals and other organisms |
| ☒ | ☐ Clinical data |
| ☒ | ☐ Dual use research of concern |
| ☒ | ☐ Plants |

## Methods

| n/a | Involved in the study |
|---|---|
| ☒ | ☐ ChIP-seq |
| ☒ | ☐ Flow cytometry |
| ☒ | ☐ MRI-based neuroimaging |

## Antibodies

| | |
|---|---|
| Antibodies used | Primary antibodies:<br>Anti-tubulin (DSHB E7; 1:1000 dilution)<br>Anti-Rluc (Invitrogen PA5-32210; 1:500 dilution)<br>Anti-Fluc (Invitrogen PA5-32209; 1:2000 dilution)<br>Secondary antibodies:<br>LI-COR IRDye® 680RD Goat anti-Rabbit IgG Secondary Antibody (LI-COR Cat# 926-68071, 1:20,000 dilution)<br>LI-COR IRDye® 800CW Goat anti-Mouse IgG Secondary Antibody (LI-COR Cat# 926-32210, 1:20,000 dilution) |
| Validation | Antibodies were validated by their respective manufacturers, all of whom cited multiple publications in which the antibodies had been utilized. Further validation in our western blotting experiments followed from the determination of the molecular weights of the identified protein bands.<br>The relevant manufacturers' websites include:<br>Anti-tubulin: https://dshb.biology.uiowa.edu/E7_2<br>Anti-Rluc: https://www.thermofisher.com/antibody/product/Renilla-luciferase-Antibody-Polyclonal/PA5-32210<br>Anti-Fluc: https://www.thermofisher.com/antibody/product/Firefly-luciferase-Antibody-Polyclonal/PA5-32209<br>Goat anti-Rabbit IgG: https://www.licor.com/bio/reagents/irdye-680rd-goat-anti-rabbit-igg-secondary-antibody<br>Goat anti-mouse IgG: https://www.licor.com/bio/reagents/irdye-800cw-goat-anti-mouse-igg-secondary-antibody |

## Eukaryotic cell lines

Policy information about cell lines and Sex and Gender in Research

| | |
|---|---|
| Cell line source(s) | HEK293 (CLS Cat# 300192/p777_HEK293, RRID:CVCL_0045) is a transformed cell line derived from human (Homo sapiens) fetal kidney. |
| Authentication | Cell line used is well-established. Cell line was routinely replaced or verified by genome sequencing. |
| Mycoplasma contamination | Cell line was routinely tested for Mycoplasma contamination and was negative for these experiments. |
| Commonly misidentified lines<br>(See ICLAC register) | Cell line used is not among commonly misidentified lines. |

## Plants

| | |
|---|---|
| Seed stocks | N/A |
| Novel plant genotypes | N/A |
| Authentication | N/A |
